# Candidate Gene Expression in *Bos indicus* Ovarian Tissues: Prepubertal and Postpubertal Heifers in Diestrus

**DOI:** 10.3389/fvets.2016.00094

**Published:** 2016-10-18

**Authors:** Mayara Morena Del Cambre Amaral Weller, Marina Rufino S. Fortes, Laercio R. Porto-Neto, Matthew Kelly, Bronwyn Venus, Lisa Kidd, João Paulo Arcelino do Rego, Sophia Edwards, Gry B. Boe-Hansen, Emily Piper, Sigrid A. Lehnert, Simone Eliza Facioni Guimarães, Stephen Stewart Moore

**Affiliations:** ^1^Animal Science Department, Universidade Federal de Viçosa, Viçosa, Minas Gerais, Brazil; ^2^Queensland Alliance for Agriculture and Food Innovation, Centre for Animal Science, The University of Queensland, Brisbane, QLD, Australia; ^3^School of Chemistry and Molecular Biosciences, The University of Queensland, Brisbane, QLD, Australia; ^4^CSIRO Agriculture Flagship, Queensland Bioscience Precinct, Brisbane, QLD, Australia; ^5^School of Veterinary Sciences, The University of Queensland, Gatton, QLD, Australia; ^6^Instituto Federal de Educação, Ciência e Tecnologia do Ceará, Fortaleza, Ceará, Brazil

**Keywords:** bovine species, intraovarian factors, quantitative real-time PCR, *corpus luteum*, folliculogenesis

## Abstract

Growth factors such as bone morphogenetic proteins 6, 7, 15, and two isoforms of transforming growth factor-beta (BMP6, BMP7, BMP15, TGFB1, and TGFB2), and insulin-like growth factor system act as local regulators of ovarian follicular development. To elucidate if these factors as well as others candidate genes, such as estrogen receptor 1 (*ESR1*), growth differentiation factor 9 (*GDF9*), follicle-stimulating hormone receptor (*FSHR*), luteinizing hormone receptor (*LHR*), bone morphogenetic protein receptor, type 2 (*BMPR2*), type 1 insulin-like growth factor receptor (*IGFR1*), and key steroidogenic enzymes cytochrome P450 aromatase and 3-β-hydroxysteroid dehydrogenase (*CYP19A1* and *HSD3B1*) could modulate or influence diestrus on the onset of puberty in Brahman heifers, their ovarian mRNA expression was measured before and after puberty (luteal phase). Six postpubertal (POST) heifers were euthanized on the luteal phase of their second cycle, confirmed by corpus luteum observation, and six prepubertal (PRE) heifers were euthanized in the same day. Quantitative real-time PCR analysis showed that the expression of *FSHR, BMP7, CYP19A1, IGF1*, and *IGFR1* mRNA was greater in PRE heifers, when contrasted to POST heifers. The expression of *LHR* and *HSD3B1* was lower in PRE heifers. Differential expression of ovarian genes could be associated with changes in follicular dynamics and different cell populations that have emerged as consequence of puberty and the luteal phase. The emerging hypothesis is that *BMP7* and *IGF1* are co-expressed and may modulate the expression of *FSHR, LHR* and *IGFR1*, and *CYP19A1*. *BMP7* could influence the downregulation of *LHR* and upregulation of *FSHR* and *CYP19A1*, which mediates the follicular dynamics in heifer ovaries. Upregulation of *IGF1* expression prepuberty, compared to postpuberty diestrus, correlates with increased levels *FSHR* and *CYP19A1*. Thus, *BMP7* and *IGF1* may play synergic roles and were predicted to interact, from the expression data (*P* = 0.07, *r* = 0.84). The role of these co-expressed genes in puberty and heifers luteal phase merits further research.

## Introduction

Ovarian activity and hormones are paramount for pubertal development and normal reproductive performance (1). Improving the reproductive performance of *Bos indicus* cattle is an industry priority in tropical and subtropical regions of the world, because of its impact on farm productivity (2). *B. indicus* breeds have a later onset of puberty (16–40 months of age), which has a negative impact on their overall reproductive performance (3–6). Although the heritability of age at puberty measured by first detected *corpus luteum* (CL) has been reported to be moderate (0.52–0.57) (7), the phenotypic identification of animals that undergo puberty at an early age is expensive. Enhancing our comprehension of ovarian genes and their interactions involved in bovine puberty could have practical implications in the animal breeding context.

The regulation of ovarian activity is an integrated process that involves FSH and LH, their receptors, ovarian steroids, and intraovarian factors (8). Some of the most important intraovarian factors are members of the transforming growth factor-beta (*TGFB*) superfamily, such as bone morphogenetic proteins (BMPs) 6, 7, and 15 (*BMP6, BMP7*, and *BMP15*) and two isoforms of TGFB (*TGFB1* and *TGFB2*), which are expressed by ovarian somatic cells and oocytes in a stage-specific manner throughout folliculogenesis (9, 10). These genes function as local regulators of ovarian follicular development and subsequently affect fertility (11–13). Experiments in mice indicate that the different isoforms of *TGFB* are responsible for diverse physiological functions (14, 15). An *in vivo* study showed that *BMP7* promotes the “recruitment” of primordial follicles into the growing follicle pool while inhibiting progesterone production and ovulation (16). Similar to *BMP7, BMP15*, and *BMP6* are part of a group of luteinization inhibitors (17). *In vitro, BMP7* induced expression of follicle-stimulating hormone receptor (*FSHR*) mRNA in human granulosa cells (18). In contrast, *BMP15* suppressed *FSHR* and luteinizing hormone receptor (*LHR*) expression (19). Given the discussed roles affecting ovulation, these intraovarian factors could modulate or influence the onset of puberty that leads to the first ovulation event.

Growth differentiation factor 9 (*GDF9*) gene expression is relevant for oocyte competence, and *GDF9* follows a similar expression pattern to *BMP15* in cows stimulated with FSH treatment (20). The relevance of *GDF9* expression in ovarian tissues of peripubertal *B. indicus* heifers is unclear, but its link to *BMP15* and FSH pathways merits investigation.

The insulin-like growth factors (IGFs) system plays a key role in follicular development and female fertility (21–23). Insulin-like growth factor 1 (*IGF1*) and insulin-like growth factor 2 (*IGF2*) have been reported to act in synergy with gonadotropins *LH* and *FSH* to stimulate growth and differentiation of ovarian follicles and subsequent synthesis and secretion of estradiol and progesterone production (24–27). Moreover, experiments in cattle (28, 29) showed that *IGF2* was the main intrafollicular IGF ligand regulating follicular growth and highly expressed in theca cells. All of these previous reports suggest that the IGFs and *TGFB* superfamily genes may have different roles in ovarian activity, related to the cycle phase and possible to endocrine regulation of puberty.

Estrogen produced by ovarian tissue in relevant for GnRH release from the hypothalamus and the expression of its receptor, *ESR1*, has been associated with puberty in mice (30). In cattle, *ESR1* expression is required for normal follicular development and follicular dominance (31). Given the relevance of steroid hormones for pubertal development and ovarian function, key steroidogenic enzymes such as cytochrome P450 aromatase (*CYP19A1*) and 3-β-hydroxysteroid dehydrogenase (*HSD3B1*) were also targeted in this study.

So far, studies have focused on the endocrine regulation of the hypothalamic–pituitary–ovarian system for onset of puberty (32–34). Information about specific changes in ovarian gene expression-associated specific cycle phases and puberty is sparse. Thus, the aim of the present study was to elucidate the expression pattern of candidate genes, such as *ESR1, GDF9, FSHR, LHR, BMPR2, TGFB1, TGFB2, BMP15, BMP6, BMP7, IGF1, IGFR1, IGF2, CYP19A1*, and *HSD3B1* in pre- (PRE) and postpubertal (POST) heifers in the diestrus phase. Evidence of differential expression will help to support or disprove the hypothesis that these genes modulate or are influenced by the presence of circulating progesterone and the onset of puberty.

## Materials and Methods

### Animal Management and Puberty Observation

Management, handling, and euthanasia of animals were approved by the Animal Ethics Committee of The University of Queensland, Production and Companion Animal group (certificate number QAAFI/279/12). Twenty Brahman heifers, which were not pedigree animals, but had a characteristic *B. indicus* phenotype and were typical beef industry animals, were sourced as young weaners born at the same season (<250 kg) and kept at grazing conditions, from two commercial herds in Queensland, Australia. After being sourced, heifers were kept at the Gatton Campus facilities of the University of Queensland; they were all under the same conditions and pasture based diet until project end. Precise day of birth information was not available for these heifers as they were sourced from industry; the effect of age differences in pubertal development is possible but could not be tested in this experiment.

Heifers were examined every 2 weeks for observation of pubertal development, from October 2012 to May 2013. Ovarian activity was observed using ultrasonography [HS-2000(VET), Honda Electronics Inc.]. Pubertal status was defined by presence of a CL observed with the ultrasound (7). Euthanasia plans were based on date of the first CL observation. Six heifers were chosen to be prepubertal (PRE) and six heifer POST. The POST heifer when identified was then paired with PRE heifer which was randomly drawn from the remaining animals and processed on the same day. The animals were weighted, and the body condition (BCS) was measured. POST heifers were euthanized 23 days, on average, after observation of the first CL. POST heifers were euthanized on the luteal phase of their second estrous cycle, confirmed by CL presence on ovarian tissue post euthanasia. Euthanasia was carried out by stunning with a non-penetrating captive bolt followed by exsanguination. Concentrations of progesterone were measured with a radioimmunoassay (RIA) from blood samples collected at exsanguination, to confirm that POST heifers had a functional CL, which was observed at euthanasia. RIA was performed by the Laboratory for Animal Endocrinology of the University of Queensland (Dr. Stephen Anderson). Plasma progesterone concentrations were measured by RIA as described by J. D. Curlewis, M. Axelson, and G. M. Stone (35) with the difference that progesterone antiserum C-9817 (Bioquest, North Ryde, NSW, Australia) was used. Extraction efficiency was 75%, and the values reported herein were not corrected for these losses. The sensitivity of the assay was 0.1 ng/ml, and the intra- and inter-assay coefficient of variation (CV) was 5.0%.

Post-euthanasia, left and right ovaries were harvested and preserved by snap freezing in liquid nitrogen, then kept at −80°C until RNA extractions were carried out. In total, 24 ovaries (2 × 6 PRE and 2 × 6 POST heifers) were processed separately for RNA extraction and quantitative real-time PCR (qRT-PCR) measurements.

### RNA Extraction and Quantitative Real-Time Transcription PCR Analysis

Prior to RNA extraction, the whole ovary tissue was ground under liquid nitrogen to form a powder of which 25 mg was used for RNA extractions; left and right ovaries were kept separately. Total RNA was isolated separately from 25 mg of the homogenized sample of left and right ovaries from PRE and POST heifers, using Trizol method (Life Technologies, Inc.). The total RNA was resuspended in RNase-free ultrapure water and stored at −80°C until further use. RNA concentrations were measured by Nanodrop ND-1000 spectrophotomer (Thermo Fisher Scientific, Wilmington, DE, USA) with an optimal 260/280 ratio between 1.8 and 2.1. Intact 28S and 18S rRNA subunit integrity was assessed by Bioanalyser (RIN 6.9 or above for all samples).

Reverse transcription was performed using GoScript Reverse Transcription System (Promega) and oligo (dT) primers (Invitrogen). The reactions were performed with 6 μg of total RNA and 2 μl of 50 μM oligo (dT)23VN primer, following the manufacturer’s recommended protocol. The cDNA concentrations from the samples were estimated on a Nanodrop ND-1000 spectrophotomer (Thermo Fisher Scientific). Finally, the single-stranded cDNA samples were stored at −20°C until analysis by qRT-PCR.

Quantitative real-time PCR reactions were performed in triplicate using SYBR^®^ Select Master Mix (Applied Biosystem) following the manufacturer’s instructions in a ViiA™ 7 Real-Time PCR System (Applied Biosystem). The CV of Ct values from replicates within each sample was low <3% indicating acceptable accuracy and reproducibility (not shown). The oligonucleotide primers used for the reactions were designed using PrimerQuest software provided by Integrated DNA Technologies, Inc. from *Bos taurus* sequences available in GeneBank database. In the present study, glyceraldehyde 3-phosphate dehydrogenase (*GAPDH*) was used as an internal control gene because it was stably expressed in our study and previously in dairy cattle (36). The list of primer sequences used for each target gene is listed in Table 1.

**Table 1:** **Gene names, primer pair sequences, annealing temperatures, and amplification efficiencies of each target**.

Gene^a^	GenBank accession no.	Primer sequence (5′–3′)	Amplicon (bp)	Efficiency (%)
*ESR1*	NM_001001443.1	F-CTCTCTGCCTTTGCTACCTTAC	114	98.87
		R-CCCGGGTCACAAATAGCTAAA		
*FSHR*	NM_174061.1	F-CATGCTCATCTTCACCGACTT	112	99.64
		R-GACCAGGAGGATCTTTGACTTG		
*LHR*	NM_174381.1	F-GAGTGGCTGGGATTATGACTATG	127	99.87
		R-CAGCCAAATCAGGACTCTAAGG		
*BMPR2*	NM_001304285.1	F-GTACCGGCATGACCACTATATC	120	99.74
		R-AGTCTTCTTCGGTCAGTTGTAAG		
*TGFB1*	NM_001166068.1	F-TGCTTCAGCTCCACAGAAA	149	99.74
		R-GTATCCAGGCTCCAGATGTAAG		
*TGFB2*	NM_ 001113252.1	F-CACGAATGGCTCCACCATAA	127	98.67
		R-AGCGTGCTTCTAGTTCTTCAC		
*GDF9*	NM_174681.2	F-GCATTCCCTCCACCCTAAA	113	99.89
		R-GGTGACGGGACAATCTTACA		
*BMP15*	NM_ 001031752.1	F-GTAGTGAGGTTCGTGAGTTCTG	111	98.85
		R-TAGGGAGAGGTTTGGTCTTCT		
*BMP6*	XM_005223892.2	F-TTCTCAACGACGCCGATATG	122	98.56
		R-ACCCTCAGGAATCTGGGATAG		
*BMP7*	NM_ 001206015.1	F-ATGACAGCTCCAACGTCATC	123	98.99
		R-AGAGACCCAGGATCCAGAAA		
*CYP19A1*	NM_174305.1	F-GTGTCCGAAGTTGTGCCTAT	101	98.95
		R-GACCTGGTATTGAGGATGTGTC		
*HSD3B1*	NM_174343.3	F-ACACCAGCACCATAGAAGTG	113	98.88
		R-CTTGCTGTATGGGTATGGAGAG		
*IGF1*	NM_001077828.1	F-TCCCATCTCCCTGGATTTCT	105	98.86
		R-GGGTTGGAAGACTGCTGATT		
*IGFR1*	NM_001244612.1	F-GTATGGAGGAGCCAAGCTAAA	123	98.98
		R-GTCTTGGCCTGAACGTAGAA		
*IGF2*	NM_174087.3	F-CATTGTGGGAAGGTGTGTC	113	99.02
		R-TTACATTCTGTGGGCTGTGG		
*B2M*	NM_173893.3	F-ACCTGAACTGCTATGTGTATGG	134	99.95
		R-GTGGGACAGCAGGTAGAAAG		
*GAPDH*	NM_001034034.2	F-GATGCTGGTGCTGAGTATGT	113	99.96
		R-GCAGAAGGTGCAGAGATGAT		

*^a^Gene symbol: ESR1, estrogen receptor α; FSHR, follicle-stimulating hormone receptor; LHR, luteinizing hormone receptor; BMPR2, bone morphogenetic protein receptor, type 2; TGFβ1, transforming growth factor beta 1; TGFβ2, transforming growth factor beta 2; GDF9, growth differentiation factor 9; BMP15, bone morphogenetic protein 15; BMP6, bone morphogenetic protein 6; BMP7, bone morphogenetic protein 7; CYP19A1, cytochrome P450 aromatase; HSD3B1, 3-β-hydroxysteroid dehydrogenase; IGF1, insulin-like growth factor 1; IGFR1, type 1 insulin-like growth factor receptor; IGF2, insulin-like growth factor 2; B2M, beta-2-macroglobulin; GAPDH, glyceraldehyde-3-phosphate dehydrogenase*.

Prior to performing qRT-PCR, the amplification efficiency and optimal primer concentration were determined for each gene using serial dilution of cDNA. For this purpose, four concentrations of cDNA (0.625, 2.5, 10, and 40 ng/reaction) and two primer dilutions (100, 200 nM) were tested. The PCR efficiencies for all primers pairs were obtained using the formula *E* = 10^(−1/slope)^ × 100, where *E* is efficiency and slope is the gradient of dilution series in the linear phase. These results are summarized in Table 1, where *E* is represented as a percentage [%*E* = (*E* − 1) × 100]. Samples were amplified separately in triplicate using a ViiA™ 7 Real-Time PCR System (Applied Biosystem, Foster City, CA, USA) with the following amplification program: an initial step at 95°C for 10 min, a second step of 40 cycles of 95°C for 20 s, and a final extension step at 60°C for 30 s. At the end of each reaction, a denaturation curve was plotted to assure that each reaction produced a single fragment, that is, the curve contained only one dissociation peak.

### Statistical Analysis

Progesterone, body weight, and condition score data were analyzed in a completed randomized design using SAS (version 9.1.3), and means were compared by the *t*-test to the level of 5%. Statistical analysis of Ct data was performed using %QPCR_MIXED macro developed in SAS (version 9.1.3) (https://msu.edu/~steibelj/JP_files/QPCR.html) developed to generate codes in SAS PROC MIXED suitable to analyze data from qRT-PCR, assuming independent random effects for reference and target genes in each biological replicate (37). The following model was used:

$$y_{\mathrm{gikr}}={\mathrm{TG}}_{\mathrm{gi}}+C_{\mathrm{gik}}+D_{\mathrm{ik}}+e_{\mathrm{gikr}}$$

where, *y_gikr_* corresponds to the Ct value obtained from the thermocycler software for the *g*th gene (reference or targets) from the *r*th well, which corresponds to the *k*th animal in *i*th physiological state (PRE or POST); TG*_gi_* is the effect of the *i*th physiological state on the expression of gene g; ${\text{C}}_{\mathrm{gik}}\sim \text{N}\left(0,\sigma_{\text{C}}^{2}\right)$ is the gene-specific random effect of the *k*th animal; ${\text{D}}_{\mathrm{ik}}\sim N\left(0,\sigma_{\text{D}}^{2}\right)$ is the sample-specific random effect (common to reference and target genes); and ${\text{e}}_{\mathrm{gikr}}\sim N\left(0,\sigma_{\text{e}}^{2}\right)$ is the residual term.

The relative expression was estimated using ΔCt method (target gene Ct – GAPDH gene Ct) as previously reported (38), where Ct is the PCR cycle number at which the fluorescence generated within a reaction crosses an arbitrary threshold. For each target gene, the comparison of gene expression between physiological state (PRE or POST) was performed by CONTRAST statement of the GLM procedure (SAS software) using Student’s *t*-test to the level of 5%. The “estimates” generated by CONTRAST analyses were used to estimate fold-change (relative expression) for pair-wise contrast of interest and were obtained by using 2^−(Estimate)^. The contrast between PRE versus POST heifers (both ovaries) was analyzed considering the average of Ct values from right and left ovary of each heifer for each gene. The other two contrasts were: (1) both ovaries from PRE versus only ovaries with CL from POST heifers and (2) ovaries from POST heifers without CL versus ovaries from POST heifers with CL. These two other contrasts should help to elucidate if differences in gene expression observe are related to the local presence of the CL itself or not (are more generally related to PRE versus POST processes). Once the efficiency (*E*) of the qRT-PCR reaction was close to 100%, one PCR cycle of difference between two samples means twice as much expression in the first sample in comparison with the second.

Pair-wise correlations between gene expression values were calculated, and its significance was tested using R software for analyses. Significant correlations were interpreted as predicted gene interactions.

## Results

### Serum Progesterone Concentrations, Body Weight, and Condition

Average serum concentration of progesterone for the PRE and POST *B. indicus* Brahman heifers were 0.4 ± 0.2 and 2.0 ± 0.7 ng/ml, respectively (*n* = 6 per group). The difference in progesterone concentration corresponds to the observation of CL in POST heifers. Associated data collected prior to euthanasia of PRE and POST heifers indicated no significant difference in body weight or body condition score between PRE and POST heifers. Body weight averages were 338 ± 54.17 and 362.6 ± 38.62 kg (*P* = 0.38), and condition score averages were 3.5 ± 0.44 and 3.75 ± 0.41 (*P* = 0.18), respectively.

### Ovarian Gene Expression

Ovarian gene expression was measured with real-time quantitative PCR in three contrasts: (1) average gene expression from both ovaries PRE versus POST heifers in diestrus; (2) average expression of PRE ovaries versus only ovaries with a CL in POST heifers in distrus; and (3) ovaries with CL versus ovaries without using only POST heifers in diestrus.

**Figure 1:**
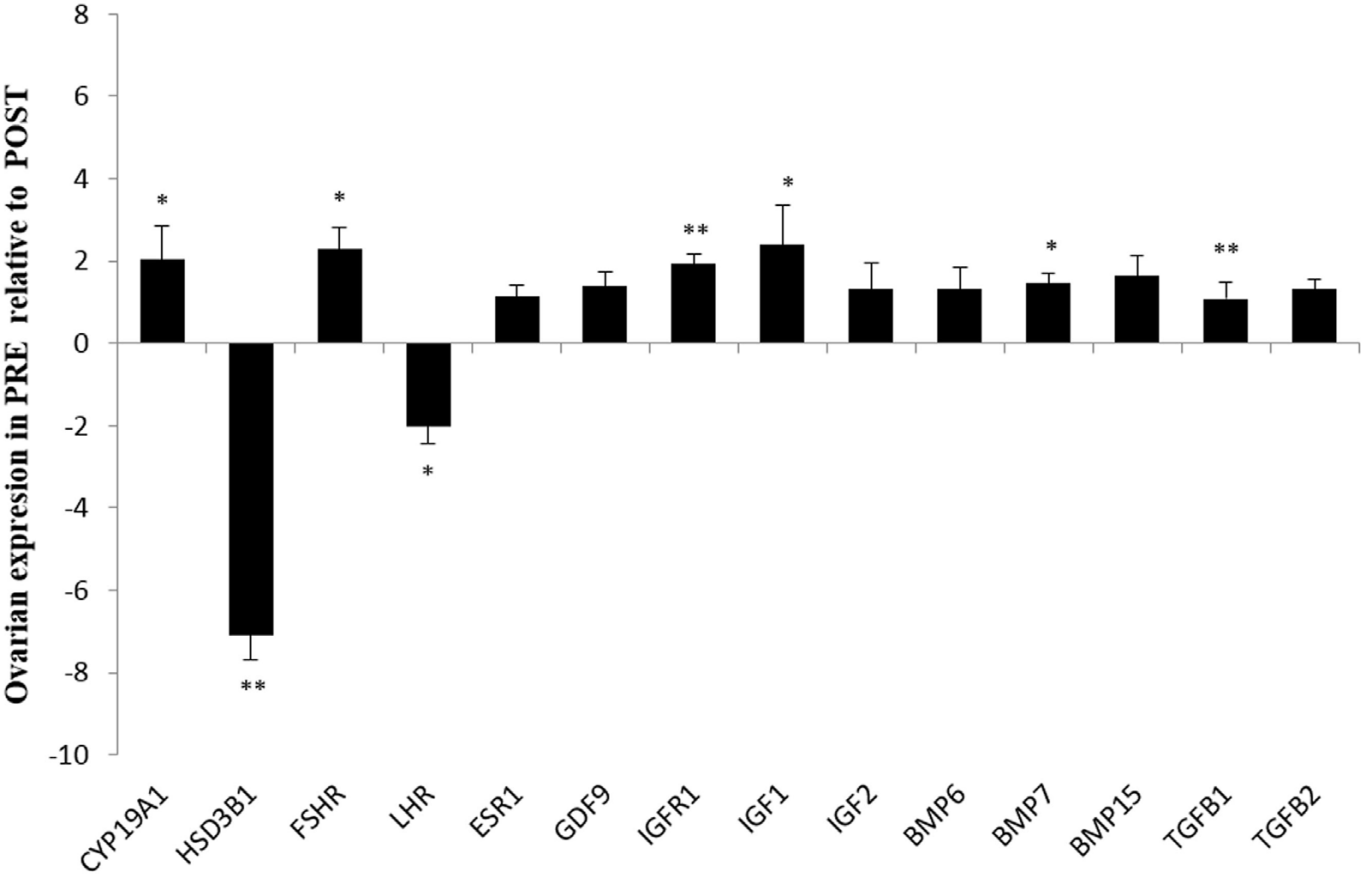
**Relative expression values for each candidate gene in both ovaries from prepubertal (PRE) heifers versus both ovaries from postpubertal (POST) heifers**. Relative expression value is expressed as the least square means ± SEM of 2^−(Estimate)^. Bars above the origin mean higher expression in PRE compared to POST. *P < 0.05; **P < 0.01.

In the contrast between PRE versus POST heifers (both ovaries), *FSHR* (*P* < 0.05), *BMP7* (*P* < 0.05), *IGF1* (*P* < 0.05), *TGFB1* (*P* < 0.01), and *IGFR1* (*P* < 0.01) expression were greater in PRE heifers, whereas *LHR* (*P* < 0.05) expression was lower in PRE heifers (Figure 1). The abundance of key steroidogenic enzyme genes, such as *CYP19A1* (*P* < 0.05) was greater in PRE heifers, whereas *HSD3B1* mRNA expression was greater (*P* < 0.01) in POST heifers.

**Figure 2:**
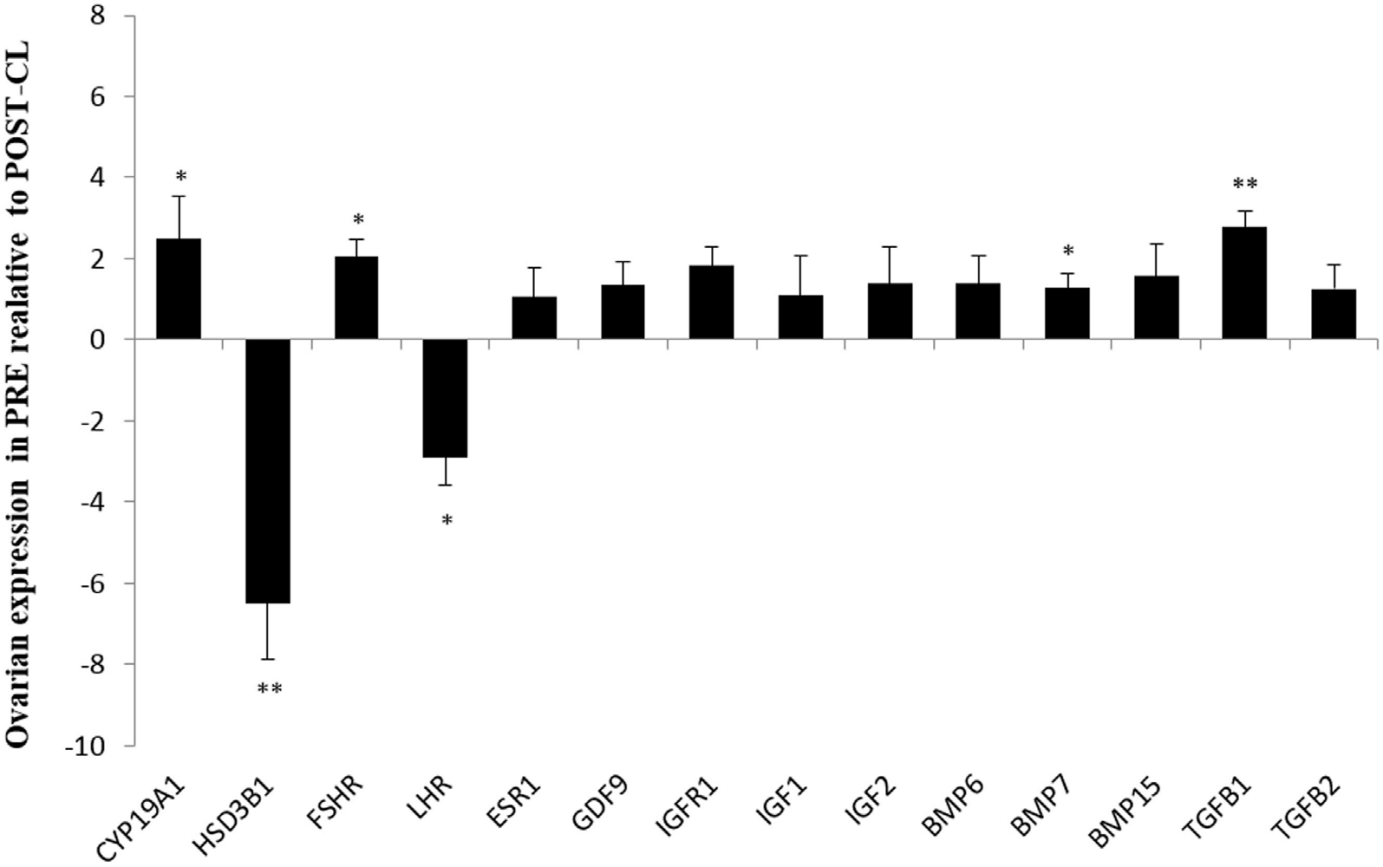
**Relative expression values for each candidate gene in both ovaries from prepubertal (PRE) heifers versus only the ovary with corpus luteum of the postpubertal (POST-CL) heifers**. Relative expression value is expressed as the least square means ± SEM of 2^−(Estimate)^. Bars above the origin mean higher expression in PRE compared to POST with CL. *P < 0.05; **P < 0.01.

In the contrast between both ovaries from PRE versus only ovaries with CL from POST heifers, *FSHR* (*P* < 0.05), *CYP19A1* (*P* < 0.05), and *TGFB1* (*P* < 0.01) expression was greater in PRE heifers, whereas expression of *LHR* (*P* < 0.05), and *HSD3B1* (*P* < 0.01) were greater in POST heifers’ ovaries with CL (Figure 2).

In the third and last contrast, we did not observe any differential gene expression between ovaries with a CL versus the contralateral ovary (without CL) from POST heifers (not shown).

Pair-wise correlations predicted four gene–gene interactions from co-expression data. The predicted significant correlations were between: *LHR* and *HSD3B1* (*P* < 0.01, *r* = −0.97); and *HSD3B1* and *CY19A1* (*P* = 0.03, *r* = −0.90). Correlations between *IGF1* and *BMP7* (*P* = 0.07, *r* = 0.84); *IGF1* and *IGFR1* (*P* = 0.10, *r* = 0.79) were high (*r* > 0.79) but not statistically significant (*P* > 0.05 and <0.10). Other pair-wise correlations between gene expression values were low and not significant (*P* > 0.1).

## Discussion

This study has demonstrated that the diestrus phase of pubertal heifers is associated with changes in ovarian gene expression of gonadotropin receptors (*FSHR* and *LHR*), key steroidogenic enzymes (*HSD3B1* and *CYP19A1*) and important intraovarian factors and their receptor, such as *BMP7, TGFB1*, and *IGFR1*. The regulation of ovarian activity is determined by coordinated action between FSH and LH with their receptors, ovarian steroids, and intraovarian factors (8). Genes of the TGFB superfamily, such as *GDF9, BMP6, BMP7, BMP15, TGFB1, and TGFB2*, and their cell receptors are known paracrine and autocrine modulators of ovarian function and fertility (9–13). The IGF system plays a key role in follicular development and is an additional important intraovarian factor involved in pubertal development (24–27, 39). Our results fit with current theory and point to specific intraovarian factors relevant to the diestrus phase of pubertal heifers in *B. indicus*.

We did not detect differential gene expression between ovaries from POST heifers with a CL versus the contralateral ovary (without a CL), which could confirm the systemic effect of circulating progesterone on ovarian dynamics. Therefore, we focused on the differences between PRE and POST heifers. In the present study, the significant differences in ovarian gene expression between two physiological states, PRE and POST (luteal phase), are likely related to onset of puberty and influenced by progesterone signaling. It is important to note that the comparison is between PRE heifers, which had never experienced a luteal phase and the related progesterone signaling, while the POST heifers were in their second diestrus with the corresponding levels of progesterone being produced by CL. Future research examining the other phases of the cycle in POST heifers would complement our findings.

We identified greater expression of *FSHR* in the ovaries of PRE than POST heifers in diestrus, which could be related to follicular waves characterized by synchronous development of groups of growing follicles, one of which would became dominant and achieved the greatest diameter, suppressing the growth of the other smaller subordinate follicles. Transrectal ultrasonography has detected the sequential growth and regression of large follicles in heifers, near the time of first ovulation (40). FSH and its receptor FSHR play an important role in follicle progression from the primary to the advanced stage of follicular development (41). Studies reported the expression of *FSHR* on granulosa cells in primary and secondary follicles, which is evidence for the role of FSH in follicular development at early stages (41, 42). Our findings are further supported by previous studies that demonstrated an increase in FSH binding sites in the rat ovary during the PRE period (43, 44). The lower expressions of *FSHR* in POST heifers in diestrus is in accord with the higher progesterone concentrations during luteal phase because progesterone exerts a negative feedback inhibition on GnRH and suppress further follicular growth and maturation (45).

The POST heifers in diestrus exhibited greater *LHR* mRNA expression than PRE heifers. These animals are in luteal phase of their estrous cycle, and it is well know that LH has essential role for maintenance and normal function of the CL (46). Therefore, LHR signaling is likely to play has an essential role in suppressing ovarian activity in the remainder of the diestrus ovary.

The BMPs are important for the regulation of follicular development, ovulation, and CL morphogenesis (12, 16, 19). Interestingly, in the present study, no significant differences in mRNA abundance of *GDF9, BMP15, BMP6 TGFB2*, and their receptor *BMPR2* were observed between the PRE versus POST contrasts. Although these genes are known as modulators of mammalian folliculogenesis and were therefore selected for investigation, it seems they are not involved with the changes in follicular dynamics observed in this contrast of PRE versus POST (luteal phase). The specific BMP associated with diestrus and pubertal development seems to be BMP7.

In the PRE versus POST (luteal phase) contrast, *BMP7* was differentially expressed. Our findings demonstrated higher abundance of ovarian *BMP7* in PRE than in POST heifers, which may be associated with the different physiological phases represented in this contrast. The ovaries of PRE heifers are likely to be constantly initiating in follicular recruitment. The recruitment stage is characterized by the presence of a cohort of growing follicles at the beginning of the follicular wave. Our finding is consistent with previous studies in mice and human showing that BMP7 promoted the recruitment of primordial follicles into the growing follicles and enhanced estradiol secretion when secretion of progesterone was concomitantly suppressed (9, 47, 48). Because progesterone is important in the process of ovulation (49), inhibition of progesterone production by BMP7 suggests a functional role as luteinization inhibitor delaying ovulation (50, 51). Furthermore, in an *in vitro* study, BMP7 increased *FSHR* mRNA expression in rat and human granulosa cells (18, 52), while it decreased *LHR* expression in human granulosa cells (18). In this context, the results of the present study suggest an important mechanism of *FSHR* and *LHR* regulation by BMP7 in PRE heifers. Greater ovarian *BMP7* contributes to increased FSH sensitivity of granulosa cells *via* upregulation of *FSHR* and downregulation of *LHR*, thus promoting folliculogenesis (recruitment, selection, and atresia) and suppressing ovulation (18).

We observed differential expression of key steroidogenic genes *CYP19A1* and *HSD3B1* between PRE and POST heifers. *CYP19A1* codes for the key regulator enzyme in the steroid biosynthesis pathways: P450aromatase, which catalyzes the conversion of androgens to estrogens (53). The upregulation of *BMP7* may explain the increased expression of *CYP19A1* in PRE heifers when compared to the diestrus POST heifers. An *in vivo* study demonstrated that rat ovaries treated with BMP7 had enhanced expression of *CYP19A1*, which in turn increased estradiol production, as well as increased the number of preantral and antral follicles (47, 54).

Greater expression of *HSD3B1* mRNA in POST heifers in their luteal phase could be the result of the presence of an active CL and consequent progesterone signaling. Theca- and granulosa-derived luteal cells express the enzyme HSD3B1 that converts pregnenolone to progesterone (55). In short, the greater expression of *HSD3B1* is necessary for production of progesterone, which is generally not occurring in PRE heifers.

Transforming growth factor-beta isoforms (TGFB1and TGFB2) are multifunctional regulatory molecules because it can either stimulate or inhibit proliferation, differentiation, and other critical cell functions according to species, stage of differentiation of ovarian cells, and presence of others growth factors (48, 56, 57). In rodents, TGFB1 increased proliferation of FSH-stimulated granulosa cells (58). However, in cattle (TGFB2) and sheep (TGFB1 and TGFB2), these growth factors have inhibitory effects on granulosa cell proliferation (59, 60). The effect of TGFB isoforms are species specific and might be different in *B. indicus* and *B. taurus* animals*. TGFB1* mRNA and protein expression in bovine granulosa cells decrease during progress of folliculogenesis (61). Furthermore, in cattle, TGFB1 was present in granulosa cells of earliest stage of development (early preantral and early antral follicles) but absent in larger more advanced follicles (62). We observed greater expression of *TGFB1* mRNA in PRE heifers compared to POST heifers (luteal phase), which highlights the important role of TGFB1 in modulating both granulosa cell growth and differentiation. We can speculate that the proliferation inhibition induced by TGFB1 may promote differentiation of granulosa cell in order to acquire *FSHR* and express steroidogenic enzymes, which allow the cell to be more responsive to FSH and enables secretion of ovarian steroids. An improved understanding of the mechanisms of TGFB1 in follicular growth and pubertal development in bovine ovary requires further investigation. Especially, it will be relevant to understand the interactions between BMP7 and TGFB1 in regulation of bovine folliculogenesis. To achieve a better understanding of this interaction, it will be relevant to study the expression of BMP7 and TGFB1 in all phases of the estrus cycle for POST heifers.

Intrafollicular IGF1 and IGF2 have been reported to act in synergy with the gonadotropins LH and FSH to stimulate growth and differentiation of ovarian follicles and subsequent synthesis and secretion of estradiol and progesterone production (24–27). The type 1 IGF receptor (IGFR1) mediates most of the actions of both IGF1 and IGF2 (63), and its expression increases during follicular development (64). In this current study, we observed greater expression of *IGF1* and *IGFR1* in PRE compared to POST heifers (luteal phase). Our findings suggest that IGF1, as well as BMP7, could be underpinning the increased expression of *FSHR* and *CYP19A1* mRNA found in PRE heifers ovaries. This is in agreement with a previous *in vivo* study that showed IGF1 can regulate *FSHR* gene expression and indirectly aromatase expression (65). *FSHR* expression has been shown to be severely reduced in preantral follicles of *IGF1* knockout mice, as well as aromatase expression, and was restored to wild-type expression levels after 2 weeks of exogenous IGF1 supplementation (66). Reinforcing this hypothesis an *in vivo* study showed that FSH enhanced *IGFR1* expression (65). Thus, local IGF1 creates a positive feedback loop where IGF1 enhances FSH action and FSH enhances IGF1 action through mutual receptor upregulation.

Taken together, our findings suggest that BMP7 and IGF1 may interact and regulate intrafollicular steroidogenesis and follicular response to gonadotropins during pubertal development. The emerging hypothesis is that two regulators of ovarian activity prepuberty are co-expressed: *BMP7* and *IGF1*. These two genes may play synergic roles modulating the expression of *FSHR, LHR*, and *IGFR1*, and steroidogenesis (*CYP19A1*). *BMP7* could be associated with downregulation of *LHR* and upregulation of *FSHR* and *CYP19A1*, which mediates the follicular dynamics in PRE heifer ovaries as compared to POST (luteal phase). Upregulation of *IGF1* expression in PRE compared to POST (luteal phase) correlates with increased levels *FSHR* and *CYP19A1*. Furthermore, TGFB1 likely plays important role in follicular dynamics in prepuberty. The role of these genes and predicted interactions merits further research to elucidate the molecular pathways in cattle ovarian tissue, pre- and postpuberty, especially to elucidate these genes expression in all cycle phases.

In summary, our results suggest that differential expression of ovarian genes could be related to changes in follicular dynamics and differences of gene expression levels within the cell population that formed the ovarian tissue, which had emerged as consequence of the luteal phase and puberty. The comparative expression of these genes in granulosa versus luteal cells in cattle PRE versus POST (including all cycle phases) should be the focus of further research to better understand their role in puberty. The data presenting herein are a starting point that reinforces the differentially expressed genes as relevant genes for ovarian dynamics in *B. indicus* heifers.

## Author Contributions

MW, MF, LP-N, SL, and SM participated in the design of the study. MF, LP-N, LK, JR, SE, GB-H, and EP carried the field trial and euthanasia of heifers. BV contributed to handling of tissue samples and RNA extraction. MW performed qRT-PCR experiment. MW and MK performed statistical analyses. MW, MF, SG, GB-H, SL, and SM drafted and revised the manuscript. All authors read and approved the final manuscript.

## Conflict of Interest Statement

The authors declare that the research was conducted in the absence of any commercial or financial relationships that could be construed as a potential conflict of interest.
